# Demyelination of the Optic Nerve: An Underlying Factor in Glaucoma?

**DOI:** 10.3389/fnagi.2021.701322

**Published:** 2021-11-02

**Authors:** Jingfei Xue, Yingting Zhu, Zhe Liu, Jicheng Lin, Yangjiani Li, Yiqing Li, Yehong Zhuo

**Affiliations:** State Key Laboratory of Ophthalmology, Zhongshan Ophthalmic Center, Sun Yat-sen University, Guangzhou, China

**Keywords:** glaucoma, demyelination, therapy, degeneration, neuroprotection, retinal ganglion cell

## Abstract

Neurodegenerative disorders are characterized by typical neuronal degeneration and axonal loss in the central nervous system (CNS). Demyelination occurs when myelin or oligodendrocytes experience damage. Pathological changes in demyelination contribute to neurodegenerative diseases and worsen clinical symptoms during disease progression. Glaucoma is a neurodegenerative disease characterized by progressive degeneration of retinal ganglion cells (RGCs) and the optic nerve. Since it is not yet well understood, we hypothesized that demyelination could play a significant role in glaucoma. Therefore, this study started with the morphological and functional manifestations of demyelination in the CNS. Then, we discussed the main mechanisms of demyelination in terms of oxidative stress, mitochondrial damage, and immuno-inflammatory responses. Finally, we summarized the existing research on the relationship between optic nerve demyelination and glaucoma, aiming to inspire effective treatment plans for glaucoma in the future.

## Introduction

Neurodegenerative diseases are a group of heterogeneous diseases with progressive and selective loss of neurons (Lin and Beal, 2006). These diseases have some common pathological changes such as axonal dysfunction, demyelination, and irreversible neuronal death. Glaucoma is a neurodegenerative disease characterized by chronic loss of retinal ganglion cells (RGCs) and their axons, with typical clinical manifestations including optic nerve atrophy, visual field defects, and diminution of vision (Weinreb et al., 2014; Jones-Odeh and Hammond, 2015; He et al., 2018). The data from population-based surveys showed that 60 million people suffered from glaucoma worldwide, of which 8.4 million became bilaterally blind, and 112 million people are expected to be suffering from glaucoma by 2040 (Quigley, 2011; Cook and Foster, 2012; Jonas et al., 2017). Developing countries lack efficient and low-cost early screening methods, so the diagnosis of glaucoma is usually confirmed at an advanced stage at which point the optimal treatment period is already missed. Glaucoma disease management in many developing countries needs further improvement. Although it is the leading cause of irreversible blindness worldwide and has been extensively investigated, the pathogenesis of glaucoma is still not yet fully understood (Weinreb et al., 2014). Therefore, it is imperative to explore the pathogenesis and establish an early diagnostic method for glaucoma.

Myelin sheath, a vital part of the nerve, is a multilayered glial membrane surrounding the axons of the myelinated nerve fiber in vertebrates. In the central nervous system (CNS), oligodendrocytes extend the processes to form myelin sheaths (Luse, 1956; Nave and Werner, 2014). Under physiological conditions, myelin plays a critical role in providing survival support to the axons, by maintaining connections and enabling efficient transmission of action potentials. The deterioration of the myelin sheaths and oligodendrocytes, known as demyelination, can affect multiple nerve fibers and form multiple disseminated lesions, leading to serious consequences, including nervous system dysfunctions (Baumann and Pham-Dinh, 2001). As the earliest pathological changes, destruction of the myelin sheath may be the basis of neurodegenerative diseases, such as multiple sclerosis, a typical demyelinating disease with axonal injury (Kaminska et al., 2017), or Alzheimer’s disease (Cai and Xiao, 2016). Therefore, demyelination plays a decisive role in neurodegenerative diseases (Coleman, 2005; Mitew et al., 2014).

**FIGURE 1:**
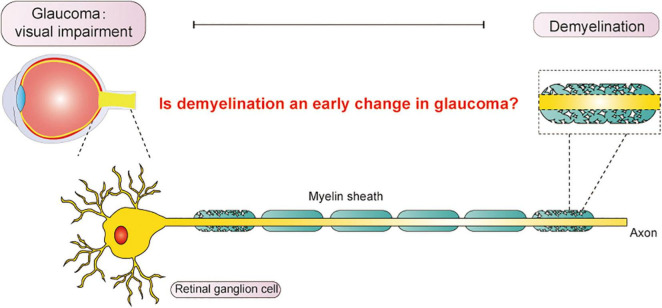
Vision impairment from glaucoma significantly influences daily life. In this article, we explored the relationship between demyelination and glaucoma, aiming to discover an early diagnosis for glaucoma in the future.

It has been proposed that demyelination occurs in neurodegenerative diseases even earlier than neuronal death and axonal dysfunction (You et al., 2019). Some researchers suggested that demyelination is a potential etiological factor of glaucoma. However, it has not been fully accepted due to the prevailing notion that demyelination occurs secondary to neuronal death and axonal dysfunction. Whether demyelination is an underlying factor in glaucoma and if the identification of demyelination could help in the early diagnosis of glaucoma remain controversial. We intend to review the possible mechanisms and manifestations of demyelination and discuss the relationships between demyelination and glaucomatous neurodegeneration that may help explore possible therapeutic directions for glaucoma in the future (Figure 1).

## The Phenomenon of Demyelination

As a type of plasma membrane, the main components of the myelin sheath are lipids and proteins. Lipids, which account for 70–75% of the dry weight of myelin, mainly contain cholesterol, galactosylceramide, and ethanolamine plasmalogen (Schmitt et al., 2015). Proteins comprise 25–30% of myelin, and the major protein components are myelin basic proteins (MBP) and proteolipid proteins (PLP) (Aggarwal et al., 2013). There is a close relationship between myelin, oligodendrocytes and axons. Therefore, morphological and functional changes in oligodendrocytes, myelin, and axons can be observed when demyelination occurs.

### Morphological Changes

#### Changes of Oligodendrocytes and Other Glial Cells

Demyelination affects the number of oligodendrocyte-lineage cells at various stages, such as the increased number of oligodendrocyte precursor cells (OPCs) after demyelination. Increased OPC can further differentiate into premyelinating oligodendrocytes and remyelinating oligodendrocytes after demyelination (Son et al., 2010; Jennings and Carroll, 2015). Other related cell types can also be affected by demyelination. For example, astrocytes are activated during the early stages of demyelination, characterized by changes in proteins such as glial fibrillary acidic protein and vimentin. During the late stages of demyelination, the number of microglial cells is increased to phagocytose the myelin sheath fragments (Rotshenker et al., 2008; Rotshenker, 2009; Son et al., 2010).

#### Changes in the Morphology of Myelin Sheaths and the Contents of Lipids and Proteins

Myelin has an unusual lipid composition — the lipid-to-protein ratio in the myelin membrane is the reverse of other cellular membranes. The molar ratio of cholesterol, phospholipids, galactolipids, and plasmalogens in myelin is approximately 2:2:1:1, respectively (Norton and Poduslo, 1973; Nave and Werner, 2014). Phospholipids are essential for the adhesive properties of myelin, and decreased phospholipid content results in phospholipid disarrangement. Based on the fact, the demyelination process can be monitored using Luxol Fast Blue (LFB), a Cu-Ti dye that interacts with phospholipids. When the optic nerve cross-sections are stained with LFB, the myelin sheaths appear as ring- or strip-shaped structures stained in blue, while other parts are transparent (Jennings and Carroll, 2015; Renner et al., 2017).

During demyelination, the contents of myelin proteins, such as MBP and myelin oligodendrocyte glycoprotein (MOG), are dramatically decreased in the myelin sheaths (Renner et al., 2017). These protein components play a critical role in the compaction of myelin sheaths. The interaction of MBP with the cytosolic membrane surfaces allows MBP to switch from an intrinsically unstructured polypeptide chain to a tightly packed protein phase, which could make a tight bond between cytoplasmic surfaces. The highly condensed cytoplasmic surfaces can be observed under EM as major dense lines (Stadelmann et al., 2019). MOG, located on the surface of myelin sheaths and oligodendrocyte processes, is crucial for the structural maintenance of the myelin sheath, especially compaction (Brunner et al., 1989). PLP is a transmembrane protein in myelin and a candidate for the tight apposition of membrane sheaths due to its hydrophilic extracellular domains (Slavin et al., 1997; Stadelmann et al., 2019). When demyelination occurs, the transcription and translation of these proteins such as MBP and MOG are affected, which could be detected by immunofluorescence and RT-qPCR (Renner et al., 2017).

#### Ultrastructural Changes in Myelin Sheaths and Axons

Morphological changes during demyelination can be evaluated using different approaches. Among them, the most intuitive and reliable method is to observe the ultrastructure and analyze the material composition of myelin sheaths by electron microscopy (EM) (Helvacioglu and Dagdeviren, 2019).

Under normal circumstances, it was observed that the myelin sheath is a stack of alternating electron-dense and electron-lucent layers, which are named the major dense line and intraperiod line, respectively (Stadelmann et al., 2019). When demyelination, vacuolation and splitting of the myelin sheath can be observed. It is widely believed that the G-ratio (ratio of the inner to the outer radius of the myelin sheath wrapped around the axon) is a parameter for assessing axonal myelination (Chomiak and Hu, 2009). An increase in the G-ratio usually means the myelin sheath thinning or the swelling of the axoplasm without affecting myelin thickness (Stikov et al., 2015). Normal axons have a normal G-ratio (0.6–0.75), while demyelinated axons lack myelin (G-ratio 1.0). Some demyelinated axons have undergone spontaneous remyelination, with thin sheaths and G-ratio greater than 0.75 (Chomiak and Hu, 2009; Berman et al., 2018; Duncan et al., 2018).

In addition, the change in axons is the breakdown of the cytoskeleton, including axoplasmic microtubules and neurofilaments, which are transformed into amorphous and granular materials. The morphological manifestations of axonal degeneration are as follows: (1) dark degeneration, where axons become dark, with dense axoplasm and miscellaneous organelles; (2) watery degeneration, where axons become pale, swollen, enlarged, and the axoplasm is either filled with an amorphous or granular material or is completely devoid of organelles (Saggu et al., 2010). Enlarged and swollen mitochondria are observed in axons, accompanied by fading cristae in the mitochondria (Coughlin et al., 2015; Thai et al., 2019). Mitochondrial dysfunction impairs mitochondrial transport along the axons and triggers necrosis of neurons (Chandrasekaran et al., 2019; Feng et al., 2021). Some RGCs have an intact cell membrane with swollen organelles; some RGCs are severely damaged with disrupted cytoplasmic organelles and electron-dense clumped nuclear remnants distributed in the cytoplasm (Narciso et al., 2001).

### Functional and Imaging Changes

Demyelination could also disrupt the integrity of the visual pathway, as reflected by reduced nerve conduction velocity and axonal damage. The action potential could not be recorded by giving a stimulation in an experimentally demyelinated sciatic nerve induced by lysophosphatidylcholine (Waxman et al., 1994). The delays in conduction along the visual system could be measured with visual evoked potentials (VEPs) (Berman et al., 2020). In addition to the morphological examination, axonal damage could be reflected by functional and imaging examination, such as diffusion tensor imaging (DTI) (Ngamsombat et al., 2020).

#### Visual Evoked Potentials

As an electrophysiological examination, visual evoked potentials (VEPs) can detect the integrity of the entire visual pathway from the retina to the visual cortex (You et al., 2015). The latency of VEP reflects the speed of signal transduction along the visual pathway, where a prolonged VEP latency implies demyelination (Heidari et al., 2019). Furthermore, the decrease in VEP amplitude indicates axon loss, inflammation, and/or demyelination (You et al., 2012).

#### Diffusion Tensor Imaging

Diffusion tensor imaging is the development and deepening of diffusion-weighted imaging, the only non-invasive examination method that can effectively observe and track the bundles of white matter fibers in the brain (Li et al., 2019). The function change can be revealed by the parameters of DTI, such as radial diffusivity (λ⊥), axial diffusivity (λ ‖), and fractional anisotropy (FA). λ⊥, the mean value of λ2 and λ3, increases when myelin sheaths are damaged due to the weakened restriction of perpendicular water molecule diffusion by myelin sheaths during demyelination. λ ‖ reflects axonal integrity. FA decreases upon axonal degeneration and myelin sheath damage (Xu et al., 2013).

Visual evoked potential and diffusion tensor imaging have been used to screen for glaucoma in high-risk groups. Recently, several studies have investigated the utility of DTI in the diagnosis of glaucoma, and it has been suggested that the parameters of DTI in the optic nerve and brain may be sensitive and reliable biomarkers for glaucoma evaluation (Li et al., 2019; Schmidt et al., 2019; Nucci et al., 2020). However, compared to IOP screening or visual field examination, VEP and DTI are relatively expensive and inconvenient, especially when the relationship between demyelination and glaucoma is unknown.

## Mechanisms of Demyelination

**FIGURE 2:**
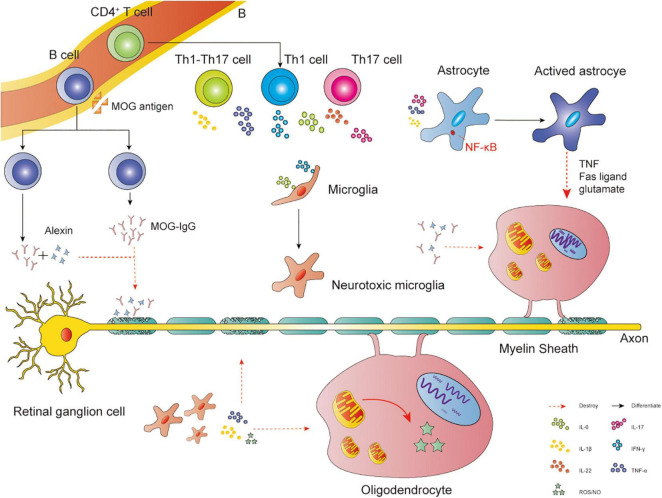
Immune and inflammatory injury. Interactions between glial cells and self-reactive lymphocytes in the CNS: CD4+ Th cells may differentiate into Th1, Th17, and Th1-Like Th17 cells. The main cytokines released by Th1 cells are IFN-γ and TNF-α, Th17 cells are IL-22 and IL-17, Th1-Like Th17 cells are IL-17 and IFN-γ. TNF-α, IL-1β, and IL-17 could activate astrocytes via NF-κB, leading to the apoptosis of oligodendrocytes via Fas ligand, TNF, and the release of NO or glutamate. IL-6 and IFN-γ could transfer microglia into neurotoxic microglia, producing NO, ROS, IL-1β, and TNF-α to attack oligodendrocytes. Pathogenic autoantibody and complement system could also play a role in demyelination. MOG antigen leaks into the peripheral blood, then transfers B cells into MOG-specific B cells, producing MOG-IgG. The antibody disrupts the interaction between MOG proteins and MBP proteins, causing demyelination. Meanwhile, MOG could directly activate the classical pathway of the complement cascade, exerting a cytotoxic effect.

There has been much discussion about the mechanism of demyelination. Here, we highlight three key points: (1) Mitochondrial dysfunction in oligodendrocytes. Such dysfunction leads to impaired synthesis of lipid, a major myelin component (Schoenfeld et al., 2010). (2) Increased oxidative stress in oligodendrocytes. Reactive oxygen free radicals or reactive nitrogen free radicals may play a potential role in oligodendrocyte death (Roth and Núñez, 2016). (3) Immune and inflammatory injury (Figure 2). Interactions between T cells and glial cells could promote oligodendrocyte injury and myelin damage (Kunkl et al., 2020; Sidoryk-Węgrzynowicz and Strużyńska, 2021). Autoantibody production and complement system also play a role in demyelination (Stern and Keskin, 2008).

### Mitochondrial Dysfunction in Oligodendrocytes

Mitochondria are essential for oligodendrocyte survival and myelination. Mitochondria are semi-autonomous organelles that have their own genetic material, mtDNA. Functional defects and morphological changes in the mitochondria have been observed in neurodegenerative diseases (Lin and Beal, 2006). The nodes of Ranvier contain a large number of mitochondria, which are responsible for respiratory reactions and encode proteins involved in the oxidative phosphorylation system. In oligodendrocytes, the transcription and translation of mitochondrially expressed genes play an essential role in differentiation. Mitochondrial fatty acid oxidation transcripts are activated to synthesize cholesterol, which is required for myelination; the transcription of DBI gene, which transfers mitochondrial cholesterol to the cytoplasm, is also increased; transcription of other myelination genes such as myelin-associated oligodendrocytic basic protein and fyn proto-oncogene are also induced (Schoenfeld et al., 2010). The dysfunction of mtDNA reduce the transcription and translation of these genes, leading to demyelination. Mitochondrial DNA double-strand breaks are also a risk factor for oligodendrocyte cell death, which then cause secondary damage such as demyelination (Madsen et al., 2017). Other research groups have also demonstrated that mitochondria are crucial metabolic factors that affect oligodendrocyte myelination.

### Increased Oxidative Stress in Oligodendrocytes

Oxidative stress can lead to demyelination of the nervous system (Ohl et al., 2016). Oligodendrocytes have relatively low levels of antioxidant ability, extensive elaborations of membranes and high levels of iron content. All features predispose oligodendrocytes to oxidative damage. Meanwhile, the myelin sheath is a preferential target of oxidative stress because of its composition and high lipid to protein ratio (French et al., 2009). Oxidative stress is caused by an imbalance between oxidants and antioxidants, or the accumulation of reactive oxygen free radicals or reactive nitrogen free radicals (Kehrer and Klotz, 2015; Kimura et al., 2017). Oxidative stress and mitochondrial damage can promote each other, forming a vicious cycle (Mahad et al., 2015). According to a previous study, oxygen free radicals can attack DNA in oligodendrocytes, and oxidative stress can also affect mitochondrial membrane phospholipids, enzymes, protein complexes, and mtDNA (Spaas et al., 2021). Oxidative stress attacks other components, in addition to mitochondrial damage. For example, matrix metalloproteinases (MMPs) are susceptible to redox state, oxygen free radicals facilitate the transcription and translation of MMP1/2/9. As an interstitial collagenase, MMPs could breakdown the basement membrane of the blood-brain barrier and degrade MBP, leading to the invasion of immune cells and demyelination (Offen et al., 2004; Ljubisavljevic, 2016).

### Immune and Inflammatory Injury

Accumulated evidence indicates that demyelination is closely associated with immune dysfunctions. For example, some neurodegenerative diseases with demyelination, such as multiple sclerosis and Alzheimer’s disease, have been associated with immune system abnormalities (Heneka, 2017; Kunkl et al., 2020). Furthermore, T cells have been found to be involved in the degeneration of the optic nerve in glaucoma (Chen et al., 2018).

The effects of T cells on demyelination in neurodegenerative diseases have been widely recognized. CD4+ Th cells are activated by mature dendritic cells. Subsequently, dendritic cells and other costimulatory molecules promote CD4+ Th cell proliferation and differentiation. CD4+ Th cells may differentiate into Th1, Th17, and Th1-Like Th17 cells. These cells can invade the CNS and interact with microglia and astrocytes, resulting in inflammatory responses by cytokines and inhibiting the maturation of oligodendrocytes and myelin sheath (Uccelli et al., 2003; Constantinescu et al., 2011; Kunkl et al., 2020). For example, inflammatory stimuli such as TNF-α, interleukin (IL)-1β, and IL-17 could cause nuclear translocation of the nuclear factor κ-light-chain-enhancer (NF-κB). The activation of NF-κB is a central step in astrocyte activation, promoting apoptosis of oligodendrocytes via Fas ligand, TNF, and NO or glutamate release (Linnerbauer et al., 2020). Microglia could transfer into neurotoxic microglia, which presents MHCII and CD86 by stimulating IL-6 and IFN-γ. Then, they produce cytokines such as NO, ROS, IL-1β, and TNF-α to promote oligodendrocyte damage (Geladaris et al., 2021).

Pathogenic autoantibodies specific to CNS antigens produced by B cells and the activation of the complement pathway also cause demyelination. For example, inflammation may disrupt the blood-brain barrier, permitting entry of MOG antigen (Zamvil and Slavin, 2015). Then the antigen could activate CD4+ T cells and recruit MOG-specific B cells, which produce a large number of antibodies. The MOG-IgG mediates the damage of MBP, destroying the myelin sheath (Ambrosius et al., 2020). Meanwhile, MOG could directly activate the classical pathway of the complement cascade, leading to demyelination (Johns and Bernard, 1999).

## Is Demyelination Associated With Glaucoma?

Thus far, it is unknown whether demyelination is an underlying factor in glaucoma. However, we do find a correlation between the two in both basic science studies and clinical patients. Clinically, in patients with primary open-angle glaucoma, researchers have observed the delay of VEP latency and an increase of λ⊥ in the optic radiation in DTI (You et al., 2019). This phenomenon indicates that demyelination occurs in patients with glaucoma. Such relevance is also proved indirectly in other demyelinating diseases. For example, more than fifty percent of patients suffer from high IOP or glaucoma in Charcot–Marie–Tooth disease type 1, whose characterized pathology is demyelination (Laššuthová et al., 2018). Furthermore, in basic research studies, researchers used laser photocoagulation to build a chronic glaucoma model in rhesus monkeys. Transmission electron microscopy analysis provided evidence of demyelination by observing myelin swelling in the lateral geniculate nucleus (Yan et al., 2017).

Thus far, only a few studies have focused on the relationship between demyelination and glaucoma. Available references are listed in Table 1.

**TABLE 1:** The different viewpoints about the demyelination in glaucoma.

**Viewpoint**	**Research model**	**Testing index**	**Evidence**
Supporting	EAG	MBP	Decrease of MBP (Reinehr et al., 2016)
	Chronic ocular hypertension	Oligodendrocytes	Oligodendrocyte loss before RGCs (Schmidt et al., 2019)
	Acute ocular hypertension	SEM	Swelling myelin (Shen et al., 2017)
	Primary angle-closure glaucoma	DTI	Decrease of FA and increase of λ⊥ (Garaci et al., 2009; Zhang et al., 2015)
Opposing	NMDA excitotoxic injury	LFB	Intact myelin sheath (Kuehn et al., 2017)
	Secondary glaucoma: DBA/2J	G-ratio	The decrease of G-ratio (Smith et al., 2018)
	Secondary glaucoma: DBA/2J	Oligodendrocytes	Axon damage precedes myelin damage (Son et al., 2010)
	EAG	LFB	RGC loss precedes myelin damage (Kuehn et al., 2018)

### Evidence That Supports an Association Between Demyelination and Glaucoma

#### Morphological Changes of Demyelination in Glaucoma

The morphology of myelin sheath is mainly observed by immunohistochemistry (LFB staining), immunofluorescence (MBP staining) and EM. Reinehr et al. (2016) established an experimental autoimmune glaucoma (EAG) model by injecting bovine optic nerve homogenate antigens. Data showed that the pathogenesis of this model is the activation of the complement system via the lectin pathway. Activation occurred at 7 days with significant RGC loss observed at 28 days; however, changes in the myelin sheath appeared early. A rapid increase in the MBP-positive area was observed at 3 days, MBP-positive area fell back to the control values after 7 days, and a decrease in the MBP-positive area occurred at 14 days (Reinehr et al., 2016). Based on the above results, we speculate that the early changes in the myelin sheath indicate glaucoma. Consistent with these results, researchers also revealed a change in MBP in a rabbit model of ocular hypertension. The authors suggest that MBP activity is a marker of stress under incipient degeneration, during which myelin is destroyed, and loosely compacted myelin sheaths can also be observed under scanning electron microscope (SEM). However, the underlying mechanisms have not yet been carefully investigated (Shen et al., 2017).

Another morphological change of demyelination reflects as the changes of oligodendrocytes. A significant change in oligodendrocytes was observed in a chronic ocular hypertensive model established by laser photocoagulation. The key point of pathogenesis is the increase in TNF-α levels. The level of TNF-α increased rapidly at 3 days after laser photocoagulation, while the increased protein levels were maintained for at least 14 days. The oligodendrocyte loss occurred at 1 week, and a loss of RGCs occurred 2 weeks after oligodendrocyte loss (Nakazawa et al., 2006).

#### Functional Changes of Demyelination in Glaucoma

In a recent study, 20 patients with bilateral chronic primary angle-closure glaucoma were examined using DTI for axonal and myelin damage of the optic nerves and optic radiations. Compared with the control group, the patients had significantly decreased FA and increased λ⊥, and increased λ⊥ correlated with mfVEP latency. Surprisingly, an increase in λ⊥ was observed not only in the optic radiation fibers projecting to the affected visual hemifield, but also in the unaffected visual hemifield. This phenomenon indicates that myelin damage precedes axonal degeneration (Garaci et al., 2009; Zhang et al., 2015).

#### Other Circumstantial Evidence

Some experiments shows that demyelination could be a target for predicting glaucoma. Increased serum anti-MBP antibody levels are a key indicator of brain damage or demyelination (Wa̧sik et al., 2020). South Korean scientists and German scientists proved that the serum levels of anti-MBP antibodies were higher in patients with primary open-angle glaucoma (POAG) and normal-tension glaucoma (NTG). Therefore, anti-MBP antibodies in serum produced by demyelination may be a marker to distinguish between control participants and patients with glaucoma (Joachim et al., 2008; Shin et al., 2020).

Some experiments have suggested that demyelination may play a role in the pathogenesis of glaucoma. Elevated peptidyl arginine deiminase 2 and decreased arginyl methylation, which mediate protein citrullination, were detected in POAG optic nerve and glaucomatous DBA/2J mice. MBP is a major citrullinated protein in the POAG optic nerve (Bhattacharya et al., 2006). Citrullinated MBP lost the ability to maintain the compaction of myelin sheaths and gave rise to large monolingual vesicles, leading to demyelination (Boggs et al., 1997). Therefore, they hypothesized that citrullination causes changes in the dynamics of myelin components, leading to the initiation of glaucomatous neuropathy (Bhattacharya et al., 2006).

### Evidence That Contradicts an Association Between Demyelination and Glaucoma

#### Changes in the Morphology of Myelin Sheaths

Some researchers regard demyelination as a manifestation of axon damage rather than an underlying factor. Kuehn et al., verified that the main pathogenic factor in the EAG model established by S100B directly destroys the axons of the optic nerve. The first change was optic nerve degeneration at 3 days and the beginning of RGC loss at 14 days, while apparent demyelination was detected at 21 days by LFB staining (Kuehn et al., 2018). Researchers revealed that a decrease in PLP gene expression and oligodendrocyte loss must follow rather than precede axon loss in old DBA/2J mice (a secondary glaucoma model induced by genetic elements and accompanied by progressively increasing IOP). Next, they analyzed an acute glaucoma rat model; half of the axons were lost 10 days after the increase in IOP, while no loss of oligodendrocytes was observed at this time (Son et al., 2010). In this case, oligodendrocyte loss is an accompanying phenomenon. Consistent with these results, in the model animals injected with N-methyl-D-aspartate (NMDA), the RGC number was significantly decreased in the peripheral and middle regions at 3 days after injury, and damage to the optic nerves occurred at 14 days. Myelin sheaths were kept intact after injury by LFB staining (Kuehn et al., 2017).

#### Ultrastructural Changes in Myelin Sheaths and Axons

Scientists have discovered different phenomena in the same DBA/2J mouse optic nerves. A study showed that myelin sheath thickness outpaced axon diameter increase, leading to a decreased G-ratio. However, the researchers did not interpret the results as an indication of early demyelination and attributed this to the upregulation of genes controlling myelin sheath synthesis. They believed that hypermyelination in the DBA/2J optic nerves was protective (Smith et al., 2018). In conclusion, changes in myelin need to be investigated further.

### Demyelination Could Be an Underlying Factor in Glaucoma

At present, most experiments focus on an unmyelinated region of the optic nerve with only few studies focusing on the myelin sheath. Even the study of glaucoma that mentions myelin sheath only describes the morphology of the myelin sheath. There are very few in-depth studies on the relationship between demyelination and glaucoma.

Based on the summary of the above literature, there is insufficient evidence from clinical studies and translational studies. Animal models are primarily used to explore whether demyelinating is the underlying factor of glaucoma, but the results are controversial. The possible reasons are summarized as follows: (1) The different pathogenesis of the models leads to significant differences in the results. Most of the current basic studies use a single animal model, which only reflects part of the pathogenesis of glaucoma (Reinehr et al., 2016; Kuehn et al., 2017; Schnichels et al., 2021). (2) The dynamic changes of the myelin sheath especially the precise time point of demyelinating have not been comprehensively studied. Most animal experiments only detect changes in myelin sheath at the late stage, while the early changes have been overlooked (Son et al., 2010). (3) The standardized and reliable quantitation methods have not been well established. The LFB and MBP staining are the most commonly used methods because they are relatively simple and feasible. However, LFB and MBP staining is a semi-quantitative technique that does not allow absolute quantitation of the myelin sheath, leading to differences in results (Khodanovich et al., 2018; Kuehn et al., 2018).

## Conclusion and Perspectives

Vision loss from glaucoma causes significant inconvenience in daily life. The incomplete understanding of the pathogenesis of glaucoma limits our ability to alleviate injury to the optic nerve and visual field defects (Weinreb et al., 2014). Currently, reducing intraocular pressure is still considered the only efficient approach to treat glaucoma (Weinreb et al., 2014; Donegan and Lieberman, 2016). Disease progression in glaucoma patients is difficult to prevent even though the intraocular pressure can be reduced by medication or surgery (De Moraes et al., 2017). Therefore, exploration of new therapeutic directions for glaucoma is highly desirable. Prevention/repair of myelin damage has become an essential part of therapy for neurodegenerative diseases, which also shows the potential to become an underlying therapeutic approach for glaucoma.

After analyzing the existing research and issues, we found several directions worthy of discussion: (1) Whether or not demyelination happens in glaucoma patients, and where it occurs, if yes. We could use various animal models in basic experiments and establish standardized quantitative methods to explore this issue. Clinically, imaging technology needs to be further developed to examine the patients. (2) If demyelination exists, whether it is helpful for the early diagnosis of glaucoma. At present, early diagnosis relies on structural and functional evaluation, mainly through optical coherence tomography and perimetry. Further study on the changes in DTI and VEP, or related antibodies and elements in the serum such as anti-MBP antibodies, could help us screen early glaucoma patients more comprehensively and efficiently. (3) We could explore the mechanism of demyelination and screen for optic neuroprotective drugs. We would like to find the critical targets of demyelination, such as muscarinic acetylcholine receptors and sphingosine one phosphate receptors, which have been explored. We hope to screen out drugs with high specificity and minor side effects, laying the foundation for translational research and clinical application.

In future, the exploration of whether demyelination is an underlying factor in glaucoma will provide new directions for the treatment of glaucoma.

## Author Contributions

JFX, YTZ, and YQL contributed to the central idea of the review and wrote the manuscript. ZL, JCL, and YJNL helped to revise the manuscript. YHZ and YQL designed and coordinated the study, and reviewed the manuscript. All authors read and approved the final manuscript.

## Conflict of Interest

The authors declare that the research was conducted in the absence of any commercial or financial relationships that could be construed as a potential conflict of interest.

## Publisher’s Note

All claims expressed in this article are solely those of the authors and do not necessarily represent those of their affiliated organizations, or those of the publisher, the editors and the reviewers. Any product that may be evaluated in this article, or claim that may be made by its manufacturer, is not guaranteed or endorsed by the publisher.
